# Rescue of the traditional song culture of a critically endangered songbird

**DOI:** 10.1038/s41598-026-40115-3

**Published:** 2026-02-25

**Authors:** Daniel Appleby, Naomi E. Langmore, Ben Pitcher, Joy Tripovich, Richard Matkovics, Robert Heinsohn, Ross Crates

**Affiliations:** 1 Fenner School of Environment and Society, Australian National University, Acton, Canberra, ACT Australia; 2 Research School of Biology, Australian National University, Acton, Canberra, ACT Australia; 3 Taronga Institute of Science and Learning, Taronga Conservation Society Australia, NSW Sydney, Australia; 4 School of Biological Science, University of New South Wales, Sydney, Australia; 5 Taronga Operations, Taronga Conservation Society Australia, NSW Sydney, Australia; 6 School of Natural Sciences, Macquarie University, Sydney, NSW Australia

**Keywords:** Adaptive management, Cumulative cultural evolution, Captive breeding, Cultural extinction, Reintroduction biology., Ecology, Ecology, Evolution, Zoology

## Abstract

Animal cultures are learned behaviours that are maintained in populations through social learning and conformity. Global reductions in species’ populations can lead to the loss of animal cultures, which may further reduce individual fitness. The Critically Endangered regent honeyeater (*Anthochaera phrygia)* is an Australian songbird whose population is declining to the extent that individuals have insufficient contact with conspecifics to maintain their song culture. Zoo-bred males that supplement the wild population sing songs that differ from all wild birds, representing a cultural barrier that may impact their fitness post-release. Over three breeding seasons within the applied breeding system, we undertook adaptive song tutoring experiments using song playback and live tutoring to teach zoo-bred juveniles the wild song. The proportion of juveniles that learned the wild song increased from zero to 42% within three years. The full version of the wild song taught to zoo-bred males disappeared from the wild during the study, making the zoo population the only remaining source of traditional song culture. Using just two wild founders, we show how animal cultures can be restored in ex-situ populations with simple modifications to husbandry protocols. Ex-situ populations could then help maintain and restore wild animal cultures through reintroductions.

**Supplementary Information:**

The online version contains supplementary material available at 10.1038/s41598-026-40115-3.

## Introduction

To address the global biodiversity crisis, ex-situ breeding of animals for release into the wild is now an integral conservation tool^1^. The success of animal reintroductions has been the subject of much scrutiny however^2–4^. Whilst practitioners increasingly apply more rigorous approaches to the management of ex-situ populations and the decision-making process around release planning^5–7^, the success of ex-situ reintroduction programs in terms of their contribution to recovery of wild populations remains questionable^8^and their success may depend on practitioners’ consideration of natural behavioural repertoires in captive animals.

The suite of factors known to affect reintroduction success is large and growing^2,3,9^. Whilst these factors are often species-specific and linked to whether the initial drivers of population decline have been mitigated^10^, there is increasing evidence that the phenotypes and associated post-release fitness of zoo-bred animals can be impacted negatively by life in captivity^11^. This in turn can hinder the contribution of zoo-bred animals to the recovery of wild populations^12–14^.

The predominant focus of ex-situ breeding programs has traditionally been on genetic diversity and disease prevention^15,16^. Whilst both factors are fundamental for maintaining viable ex-situ populations, behavioural differences between zoo-bred and wild animals rank among the top reported issues hindering the success of reintroduction programs^9^. Manipulating behaviours to improve translocation success is an emerging tool for adaptive management of translocation programs^17^. Such manipulations have largely focused on pre-release exposure to novel-stimuli such as antipredator training, or soft-release strategies where individuals can learn and adapt behaviours to novel-environments before being released fully^18,19^. Culturally acquired behaviours that may impact translocation success have received relatively little focus in translocation planning^17^, but are increasingly being recognised as a critical component of effective conservation^20,21^.

Animal culture refers to the transmission and maintenance of learned behaviours, traditions, and knowledge within animal populations through social learning processes such as observation, imitation, and teaching^20,22^. Similar to human culture, animal cultures encompass a wide range of behaviours, including feeding techniques, communication signals, mating rituals, and tool use, which are transmitted within and across generations and may vary between different social groups or populations^20,23^. These cultural behaviours often contribute to the adaptation and survival of individuals within their respective environments and can shape social dynamics and ecological interactions within animal communities. In captivity, culture may be degraded or lost by preventing necessary social interactions and exposure to suitable adults from which juveniles learn key behaviours^11^. This is problematic where ex-situ populations contain candidate individuals for release into the wild, as culturally-acquired behaviours may be critical for post-release fitness^20^. Birdsong is one of the most well-known examples of animal culture^20,22^. Learned vocalizations in songbirds serve critical functions in mate attraction, territory establishment and defence, species recognition, and social cohesion^21^. The loss or degradation of song culture in ex-situ and/or wild populations can therefore have direct fitness consequences by reducing breeding success and limiting social integration.

The regent honeyeater (*Anthochaera phrygia*) is a Critically Endangered Australian songbird^24^whose population has declined primarily due to extensive loss and degradation of woodland habitats through agricultural clearing, dieback, and forestry practices, while remaining habitat is further threatened by climate-change driven drought and bushfire^27^. With an estimated wild population of fewer than 250 individuals^25^, zoo breeding to bolster the wild population through reintroductions and provide an insurance against further population decline is a high priority recovery strategy^26^. Supplementation of the wild population with zoo-bred birds has had limited success in arresting the population decline however^27,28^, with the majority of zoo-bred birds failing to establish a breeding territory or pair with wild mates^28,29^.

Wild male regent honeyeaters sing only one of several song types with loose geographic dialects^30^. Females of this species do not produce the full song that the males sing, and instead produce subtle calls, warbles and ‘mews’ which may be innate. The dominant wild song type within the core range of the remaining wild population in New South Wales is defined as the ‘Typical Blue Mountains’ song^30^. A severe decline in the size and density of the remaining wild regent honeyeater population is leading to loss of the species’ song culture – many young males either learn the songs of other species or sing an abbreviated version of the ‘typical Blue Mountains’ song with half the number of syllables^30,31^. The nomadic ecology of regent honeyeaters means young males encounter different heterospecific models at dispersed breeding sites, precluding convergence on a shared, species-specific song culture^30^. As population density declines and social learning opportunities diminish, song degradation likely impairs mate attraction and territorial function, potentially contributing to Allee effects^45,46^.

Zoo-bred males sing a distinct abnormal song that is different from all wild song types^30^. As passerines, young male regent honeyeaters learn their songs via exposure to and imitation of conspecific tutors^32^. The zoo population was founded through the collection of nestlings in 1995 and since then juvenile males have been ‘crèched’ together during their critical song-learning period in early life^30^. Zoo-bred regent honeyeaters have therefore never had the opportunity to learn wild regent honeyeater song culture from older conspecifics. Instead, they crystallise an adult song that resembles the warbling calls of juveniles, suggesting zoo-bred juveniles learn songs from each other in the crèche. The distinct differences between the songs of wild and zoo-bred birds may represent a significant cultural barrier to the assimilation of zoo-bred birds into wild flocks due to assortative mating^33^. Zoo-bred females prefer the familiar but aberrant songs of zoo-bred males over the unfamiliar songs of wild males^14^.

We aimed to eliminate an important cultural divide between a zoo-bred and wild animal population within an applied zoo-breeding environment by teaching zoo-bred regent honeyeaters the dominant wild song culture in the area they will be reintroduced to through song tutoring experiments and adaptive management. Song tutoring experiments occurred over three years within six different treatment groups that varied principally in tutoring method and cohort size (Table 1). At the start of the study, only two wild origin male regent honeyeaters that were recruited to the zoo population sang the Typical Blue Mountains song and were available as live tutors. The alternative tutoring approach was to use song playback, based on recordings of other wild males’ songs from the Blue Mountains population. We adaptively modified treatments in later years of the study based on the results of the previous years’ tutoring approaches. Our overall goal was to seed the dominant wild song type into the zoo-bred population culturally, such that the wild song culture can be sustained in captivity as part of routine management practice. Our secondary goal was to gain insights into the key factors determining song learning in regent honeyeaters.

## Results

**Fig. 1:**
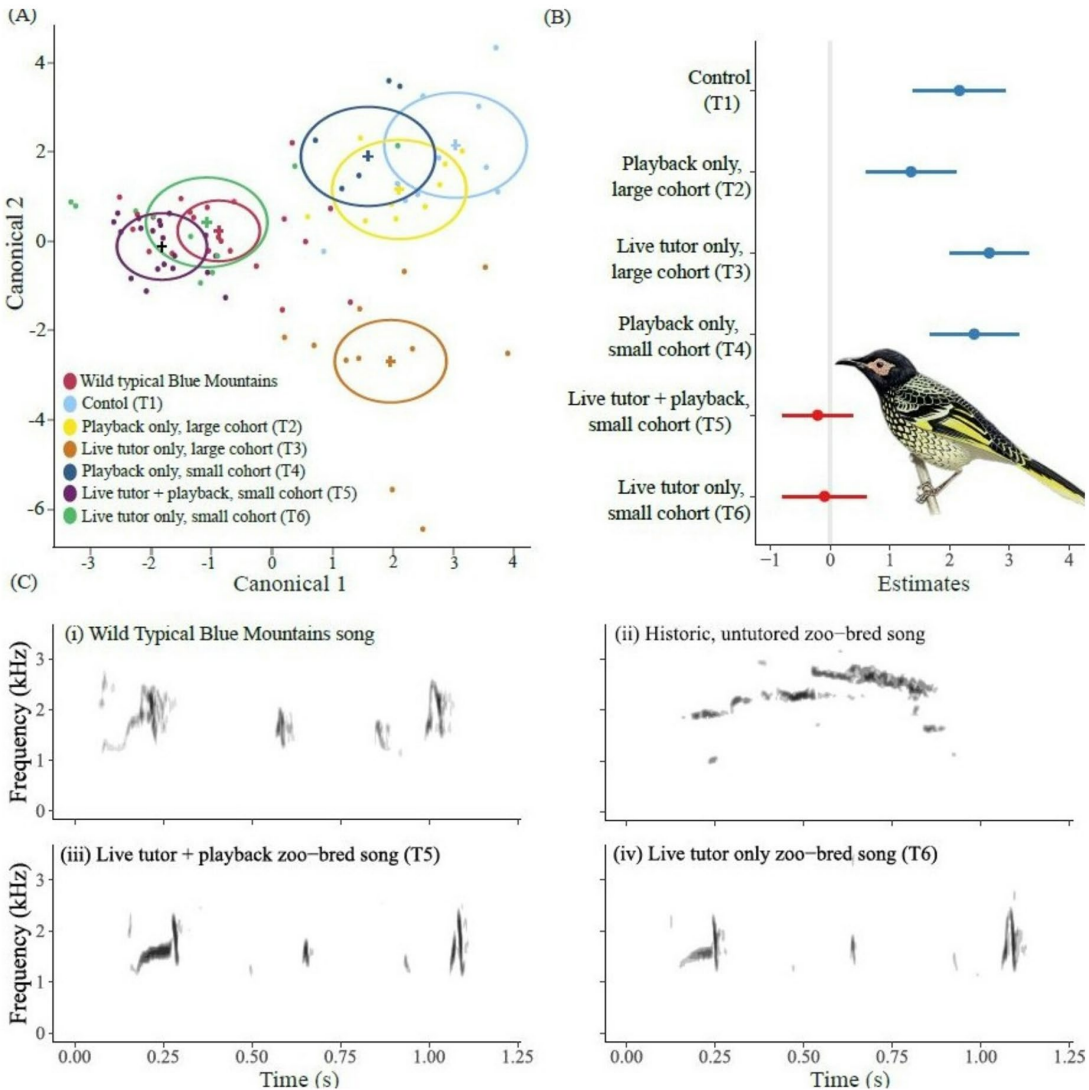
(**a**) Discriminant function analysis (DFA) of regent honeyeater songs by treatment group. DFA labels each treatment group or reference group multivariate mean with a circle corresponding to a 95% confidence limit for the mean. Groups whose songs are significantly different have nonintersecting circles. The first two discriminant functions explained over 84% of the total variance in the song samples. The model’s overall significance was supported by multiple test statistics (wilks-lambda = 0.062, f = 20.10, p = < 0.001; Pillai’s Trace = 1.83, f = 13.90 p = < 0.001); (**b**) Model estimates showing the effect of regent honeyeater song tutoring experimental treatment on the similarity of songs to the culturally dominant ‘typical Blue Mountains’ wild regent honeyeater songs based on Mahalanobis distances. Points show predictions, lines show 95% confidence intervals. Estimates in red show the two treatment groups (5 & 6) whose songs show no statistically significant difference to the typical Blue Mountains reference songs as per Table 2. (**c**) Spectrograms showing example songs of: (i) wild Typical Blue Mountains; (ii) Historic zoo-bred song; (iii) tutored zoo-bred male with live tutor and playback (Treatment 5); (iv) tutored zoo-bred male with live tutor only (small cohort- Treatment 6). See Fig. S2 for indicative spectrograms of juveniles in treatment groups 1–4.

Before the first year of the experiment, only the two wild-origin males sang the typical Blue Mountains song within the zoo population. By the end of the experiment, 32 males representing 42% of the male zoo population, which fluctuates annually depending on the number bred and the number reintroduced to the wild, sang songs that were statistically indistinguishable from the typical Blue Mountains song based on discriminant function analysis (DFA) and general linear models (GLMs, Fig. 1). We successfully developed a sustainable song tutoring protocol based on simple modifications to existing husbandry practices that will give future generations of regent honeyeaters born in captivity the opportunity to learn culturally conforming songs from older conspecifics.

During the first year of the experiment, no juveniles successfully learned the typical Blue Mountains song (Treatments one – three: control group, playback only large cohort and tutor only large cohort, Table 1; Fig. 1a). Model predictions based on Mahalanobis distances showed the average songs of males in all three groups were significantly different from the wild reference group (Fig. 1b; Table 2).

**Table 1:** Summary of the zoo-bred regent honeyeater song tutoring experimental design under an adaptive management framework showing cohorts of juveniles, their tutoring treatment, site, aviary type and cohort size. Breeding site TWPZ refers to Taronga Western plains Zoo, Dubbo; TZ refers to Taronga Zoo, Sydney. Zoo-bred birds in the tutor origin column refer to zoo-bred males that successfully learned the typical blue mountains song in previous years of the study. See Fig. S1 for description of aviary types.

Treatment group	Breeding season	Breeding site	Aviary type	No. males in cohort	No. live tutors	Tutor origin
1. Control	2020/21	TWPZ	2	9	N/A	N/A
2. Playback only, large cohort	2020/21	TWPZ	2	9	N/A	N/A
3. Live tutoring only, large cohort	2020/21	TZ	2	13	1	Wild
4. Playback only, small cohort	2021/22	TWPZ	2	4	N/A	N/A
	2021/22	TWPZ	2	5	N/A	N/A
5. Live tutoring + playback, small cohort	2021/22	TZ	1	4	1	Wild
	2021/22	TZ	1	4	1	Wild
	2022/23	TZ	1	4	1	Wild
	2022/23	TZ	1	5	1	Zoo-bred
6. Live tutoring only, small cohort	2022/2023	TZ	2	5	2	Zoo-bred
		TZ	2	5	2	Zoo-bred
		TWPZ	3	4	2	Zoo-bred

By the end of the second breeding season, no juveniles in Treatment four (playback only, small group) produced the typical Blue Mountains song, however eight juvenile males in Treatment five (live tutor + playback) learned to sing songs that closely-resembled the typical Blue Mountains song type. DFA was unable to distinguish the songs of these birds from the wild reference songs (Fig. 1a), with the GLM confirming the songs of birds in this group were not significantly different from the wild reference songs (Fig. 1b; Table 2).

Four males from Treatment group five were employed as live tutors in the third year of the experiment. Together with the two wild origin birds, these four males successfully tutored an additional 19 juveniles in year three; all within Treatment group six (live tutor only, small cohort). The songs of these birds could also not be distinguished from the typical Blue Mountains reference songs by DFA (Fig. 1a). Again, there were no significant differences between the songs of birds in Treatment six and those of the wild reference group (Fig. 1b-c). Despite having a shorter tutoring period, songs of younger juvenile males hatched later in the season were no less similar to the reference songs than those of older males from first clutches (Table S1).

**Table 2:** Model summary derived from a general linear model showing the effect sizes of treatment group in explaining the differences between the songs of zoo-bred regent honeyeaters. Models are based on the Mahalanobis distance between the mean acoustic attributes of males in each group relative to the average culturally-dominant typical blue mountains wild song. P-values in bold indicate significant differences at the *p* <.05 level. See Fig. S3 for model diagnostics.

Coefficient	Estimate	SE	t	*p*
Control (T1)	2.16	0.40	11.39	**< 0.001**
Playback only (large, T2)	1.35	0.30	−0.72	**< 0.001**
Live tutor (large, T3)	2.66	0.34	7.91	**< 0.001**
Playback only (small, T4)	2.41	0.38	6.33	**< 0.001**
Live tutoring & playback (T5)	−0.21	0.30	−0.72	0.48
Live tutoring (small, T6)	−0.10	0.36	−0.27	0.79

## Discussion

The importance of considering animal cultures in conservation programs is increasingly being acknowledged^20^. We show that it is possible to reintroduce lost wild culture into an ex-situ animal population by providing the first example of successful song tutoring in an active conservation program. Both live tutoring (small cohort) and live tutoring with playback supplementation were successful in training birds to sing songs that did not differ significantly from the reference wild song. We were able to utilise birds from previous year’s successful experiments as additional tutors to increase the rate at which the wild song culture was culturally seeded in the zoo population. Playback tutoring in both large and small tutor groups was unsuccessful and did not differ significantly from the control. Live tutoring (large cohort) alone did differ from the control but was also significantly different from the reference wild song. The results of our study, whilst limited in design by the unavoidable constraints of operating within an applied zoo-breeding facility, suggest that being able to interact with a live tutor in a social group with a low ratio of adult tutors to juveniles are the key factors influencing whether birds are able to successfully acquire the wild song culture.

In numerous bird species, tutoring via playback is observed to be less effective than instruction from a live tutor^34,35^. The understanding of avian learning processes predominantly stems from a limited selection of taxonomic groups. In this study, birds subjected solely to playback tutoring exhibited an expansion in song complexity relative to untutored, zoo-bred birds but failed to replicate accurately the target wild-type song. It has been suggested that birds exclusively exposed to playback tutoring may exhibit lower success rates in learning the reference song^35,36^. As the birds progress into the sensorimotor stage of learning, the songs of associates may provide a more enriched form of learning than the playback^36^, which could facilitate horizontal transmission of song among juveniles within the aviary. This horizonal transmission would then be compounded by the addition of younger birds throughout the experiment who may learn predominantly from older juveniles rather than the audio playback. This hypothesis aligns with the findings of Derégnaucourt and Gahr^37^, who show that within generation song learning can occur in zebra finches *Taeniopygia castanotis* where a young bird exposed to songs of a tutor can teach another bird not exposed to the tutor some elements of reference song. In that study, juvenile songs converged more closely with each other than with the initial reference tutor.

The live-tutoring only (large cohort) also failed to facilitate juveniles to accurately produce wild-type regent honeyeater songs, although the songs of juvenile males within this group were closer to the wild reference songs than those of the playback treatments. We propose a similar mechanism for this result; in this instance juvenile birds did not receive playback during the earliest stages of independence, but rather had no tutoring until the adult tutor was moved into the experimental aviary. While the exact timing of song learning in this species is unknown, we propose that the introduction of the live tutor fell between the sensory and sensorimotor stages and as such limited the amount of sensory learning opportunity for the juvenile males in Treatment three. Experiments depriving young birds of tutors have demonstrated that the absence of a tutor in critical learning phases reduces the likelihood that young birds will learn new syllables after the introduction of a tutor^38^. It is also possible that as the oldest birds were deprived of a live tutor for 2.5 months, their maladapted songs may have then been horizontally transmitted to other birds in the cohort.

The two successful treatments shared two key features: opportunities for multimodal learning from live tutors and small cohorts. There is probably a relationship between smaller cohorts and truly multimodal learning; where the live tutoring only (large cohort) presented the *opportunity* for multimodal learning, it was likely not realised due to the large ratio of students to tutors. The small cohort size may have allowed for frequent and prolonged interaction with the tutor, and this is consistent with previous research that links the number of tutors with song learning fidelity^39^. Anecdotally, we observed this occurring in real time; in the large cohort group the tutor was overwhelmed by practicing juveniles and frequent song practicing was seen between juveniles. In the small cohorts, the adult male dominated high value areas like the feeders more easily. During these interactions we observed head-bobbing and bill-snapping typically associated with the crescendo of the typical Blue Mountains song.

While both successful cohorts were not statistically distinguishable from the reference wild song, birds that only received live tutoring resembled their specific tutor more closely than the wild reference. This is perhaps due to the increased variation in reference songs available to these birds but critically, unlike the playback only cohort, the live tutoring + playback cohort had exposure to a wider range of songs, that may impact the reference template and birds own song experience consistent with the eavesdropping hypothesis^40^.

While it is possible that natural cultural drift caused the divergence over time between wild and zoo-bred regent honeyeaters, it is highly likely that hand rearing of founder juveniles caused significant cultural divergence and drift within the zoo population from the very start^14,30^. Our results show that although the control group remained the least similar of all treatments to the typical Blue Mountains song, those birds’ songs were considerably more similar to the typical Blue Mountains wild song than historical ex-situ songs. This may be reflective of the increased socialisation that a larger ex-situ population affords or may be due to changes to housing arrangements that crèche juvenile birds closer to adult, albeit ex-situ tutors.

The results of our study may have significant implications for conservation efforts. Regent honeyeaters bred in captivity have generally shown a low propensity to integrate with wild birds after being released and an even lower propensity to pair and breed^30^. Assortative mating based on differences in song culture is common amongst birds^41,42^and previous research suggests that zoo-bred female regent honeyeaters prefer the familiar songs of zoo-bred males to the unfamiliar typical Blue Mountains song produced by most wild males^14^. Our experimental design exposed female juveniles to wild song tutoring alongside males in all treatment groups, potentially influencing their subsequent mate preferences. While we did not measure female song preferences in this study, exposure to wild-type songs during development could shift female preferences toward males singing wild-type songs, potentially reinforcing rather than undermining the cultural restoration we achieved in tutored males. Future research should examine whether early exposure to wild song culture affects female mate choice in this species. Reintroducing wild songs to the ex-situ population may increase the likelihood of breeding events between reintroduced and wild regent honeyeaters^14^and increase social cohesion at the flock level^43,44^.

At the inception of this project the typical Blue Mountains song was the most common song sang in the target release site^31^. Since 2020, monitoring has shown that the typical Blue Mountains song has effectively disappeared from the wild population and has been replaced by the more simplified ‘clipped Blue Mountains’ song type that contains half the number of syllables^31^. This raises an important consideration: if current wild birds predominantly sing the simplified song, teaching zoo-bred birds the traditional complex song could theoretically reduce initial pairing success due to assortative mating preferences. However, restoring traditional song culture aligns with the broader hierarchy of conservation objectives for this species. While maximizing short-term pairing success between reintroduced and wild birds is important, the primary goals remain long-term population recovery and maintenance of functional breeding systems. The simplified song may itself reflect ongoing population decline and could perpetuate Allee effects that undermine population viability^45,46^. Moreover, as released zoo-bred birds now represent an increasingly large proportion of the wild population and are the sole repository of traditional song, reintroductions offer an opportunity to reverse cultural erosion rather than simply accommodate it. From a practical standpoint, continuously adapting tutoring protocols to match rapidly eroding wild song culture is unlikely to be feasible given the multi-year timescales of captive breeding programs and any associated cultural tutoring that may be required. Providing a stable cultural reference through traditional song tutoring creates beneficial inertia against further simplification, and our results demonstrate that even imperfect song learning produces substantially more complex and functional songs than those of untutored zoo-bred birds.

Whilst cultural drift and revolution are common features of animal populations, it is more likely that simplification of the song culture is reflective of an ongoing decline in the size and density of the wild regent honeyeater population^27^, making it increasingly challenging for cultural diversity to be maintained at the population level through social interactions^30,31^. This decline in song complexity may have downstream Allee effects that negatively impact the viability of the wild population^45,46^. The results of our study mean that zoo-bred birds are now the sole source of traditional song cultural knowledge in regent honeyeaters. As it has been shown previously that songbirds can learn songs experimentally in the wild^47^, the results of our study open up the exciting opportunity to use the reintroduction of zoo-bred birds to re-establish or reinforce the species’ traditional song culture in the wild population.

Our results highlight several questions for future research. First, determining whether song learning can begin during the nestling period, and whether early playback exposure would enhance or interfere with subsequent learning, remains unclear for this species. Second, investigating whether developmental exposure to wild songs shifts female mate preferences would clarify the long-term viability of cultural restoration in reintroduced populations. Finally, systematically varying tutor introduction timing could reveal the critical windows for sensory and sensorimotor learning in regent honeyeaters, improving our ability to optimize tutoring protocols.

As global biodiversity declines, it is likely that the erosion or loss of animal cultures will become more apparent over coming years^30^. Whilst bird song provides arguably the most prominent examples across a range of species whereby declines in population size or density can impact the persistence of culture^48^, maintaining a sufficient number and frequency of social interactions is important for conserving socially-learned behaviours across multiple domains^49^. Our study highlights the important role of experienced adults as repositories of cultural knowledge in conservation programs. It also demonstrates the importance of facilitating social interactions at all stages of the husbandry process in ex-situ breeding, to help maintain socially acquired behaviours that may have a significant bearing on post-release fitness and the overall effectiveness of reintroduction programs^11^. While the song learning timeline and processes we describe are shared across many songbird species, the specific details vary between taxa, and we encourage researchers to adapt this protocol based on the developmental trajectories and learning mechanisms of their target species. For species where wild populations are or too small or sparsely distributed to practically provide live tutors, we recommend proactive recording of vocal cultures before populations decline to critical levels; however, our results suggest that recordings alone are insufficient and conservation practitioners should prioritize maintaining access to live adult tutors even in small captive populations. Given the increasing recognition of the need to consider animal cultures in conservation^22^, we provide clear evidence that this can be achieved rapidly and at little cost within applied conservation settings through adaptive management. There is great scope for similar studies on other endangered populations s (e.g. Cirl Buntings^63^) to help reduce to the phenotypic divide between wild and zoo-bred populations^11^. This will require improved monitoring of wild animal populations to better understand the dynamics of culturally-acquired behaviours^50^, as well as increased collaboration between academics and applied conservation practitioners to facilitate research, reporting and implement evidence-based changes to zoo-husbandry and reintroduction strategy^11^. Such actions could make a significant contribution to minimising the loss of not only global biodiversity over coming decades, but also its emergent properties such as birdsong that form an intrinsic part of human culture and wellbeing.

## Methods

### Study population and tutoring protocols

Birds were born and housed in Taronga Zoo (TZ), Sydney and/or Taronga Western Plains Zoo (TWPZ), Dubbo. All birds included in this study were part of the active zoo-breeding program and therefore subject to routine husbandry protocols. Experiments needed to be incorporated in the least disruptive manner to ensure they achieved the overall aim of establishing wild song culture in the zoo population without impacting negatively the breeding capacity of the program.

During the experiments birds were housed in one of three aviary types (Fig. S3). We conducted tutoring experiments over three Austral post-breeding seasons (September - March) commencing in 2020. We implemented five treatments and one control group across the two breeding institutions in the first season and adaptively refined the treatment groups before each subsequent season based on the results of previous seasons’ experiments. See Supplementary text S1 for further information on the study population and treatment groups.

We considered two common experimental song tutoring techniques as options for teaching juvenile birds to sing the ‘typical Blue Mountains’ wild song.; live tutors and audio playback. Live tutoring has shown more success than audio-only tutoring^34,51^, however due to the rarity of this species in the wild, acquiring wild birds is a logistical and ethical challenge that is compounded by regional song differences and the presence of wild birds with abnormal songs^30^. Only two wild males recruited to the TZ breeding population in 2019 sang the ‘typical Blue-Mountains’ song and were available as live tutors in the first year of the experiment. Regent honeyeaters are probably ‘closed-ended’ song learners, with males’ songs becoming largely fixed after a period of learning and crystallisation in the first year of life^30^.

Playback tutoring has proven successful in many species^52^and is logistically feasible in the zoo-breeding setting. Wild regent honeyeater song recordings were available from previous research^30^and could be implemented with minimal disruption to the zoo’s routine husbandry activities (Supplementary text S2). The remaining years of the study were adapted based on preliminary results of the previous years, the rationale for which can be found in Supplementary text Text S3.

### Standard timeline

All treatments shared the same timeline, with juvenile males transferred from their natal aviaries to their respective experimental crèche aviaries (Fig. S1) approximately two to three weeks post-fledging (~ 30 days old). Juveniles are typically moved out of their natal aviaries at this point as fathers become territorial and aggressive towards juveniles prior to re-nesting with second or third clutches per season^53^. Juvenile males were exposed to song tutoring from the time they entered the experimental crèche until the conclusion of the breeding season when they are transferred to a larger, multi-species flight aviary^28^,. See Table S2 for details of experimental timelines. Some juveniles from second or third broods received a shorter duration of song tutoring (Range = 128–208 days, mean = 162), but all were moved into the experimental aviaries at approximately 30 days old. This reflects the typical wild song-learning environment, where juveniles from different broods join larger, mixed-age post-breeding flocks towards the end of the breeding season^54^. Table 1 summarises the five treatments and control. In summary, the experiment involved 68 birds: two wild origin males (tutors) and 66 juvenile zoo-bred males, of which four were used as live adult tutors in subsequent years (Table 1). See Supplementary materials for full details of each treatment group.

Female juveniles were housed alongside males in all treatment aviaries at approximately equal sex ratios and were exposed to the same tutoring protocols. However, as female regent honeyeaters do not sing, this study focused exclusively on measuring song learning outcomes in males.

### Acoustic environment of natal enclosures

Breeding pairs were housed in adjacent aviaries at both TZ and TWPZ. Male regent honeyeaters do not sing during active breeding or while tending to chicks^30^but non-breeding males housed in nearby single-sex groups were occasionally audible from natal enclosures, providing incidental exposure to adult vocalizations prior to fledging. We did not implement playback protocols in natal enclosures for two reasons: first, to avoid inducing territorial stress in breeding males from perceived rival songs within their territory, and second, because males cease vocal activity during the nestling period to reduce nest predation risk. Experimental tutoring protocols therefore commenced when juveniles were transferred to crèche aviaries post-fledging at approximately 30 days old.

### Playback parameters

Playback tracks consisted of songs from 25 different wild male regent honeyeaters singing the typical Blue Mountains song type (each song ~ 2 s duration). To approximate natural variation in songbird vocal activity, songs were broadcast at higher rates during peak periods (~ one song per 25 s; first four hours and last two hours of daylight) than during off-peak midday periods (~ one song per 93 s). Playback rate was doubled in the 2021–2022 season based on observations that juveniles vocalized more frequently than the initial playback rate. Tracks were broadcast from sunrise to sunset daily via Bose FreeSpace outdoor speakers mounted within aviaries. Playback was broadcast at amplitudes approximating natural song volume (55–75 dB SPL measured at 3 m from the speaker), with variation reflecting the natural range of vocal amplitudes across the 25 recorded individuals. Complete details of track composition and playback systems are provided in Supplementary Text S2.

### Song recording and editing

Juveniles’ songs were recorded in their tutoring aviaries at the end of each tutoring period (October-March; Age range: 2.5 months – seven months) before they were moved into a multi-species flight aviary in preparation for potential release to the wild. Songs of each juvenile male were recorded over two days at a typical distance of one to four metres using a Sennheiser ME66 microphone and a Zoom H4n Pro recording device (48 kHz sampling rate, 16 bit). For comparison with the songs of zoo-bred juveniles included in the tutoring experiments, we also obtained recordings of (i) ‘typical Blue Mountains’ wild birds that were made in the wild between 2015 and 2019 (which formed the playback tracks used for audio tutoring); and (ii) zoo-bred birds that had not been exposed to song tutoring either one week after release into the wild in 2017 (n = 12) or within captivity in 2019 (n = nine). These additional recordings used the methods detailed in Crates et al. (2021)^30^. We first edited individual songs (342 total, min four max 13 per individual) in Apple Garageband (v10.3.3) to trim them to include only the target individual’s song and no other background noise.

### Spectral and statistical analysis

To assess the similarity between the songs of tutored juveniles and the reference wild-type typical Blue Mountains males, we first performed semi-autonomous syllable segmentation using the open-source Python software ‘Chipper v1.0’^55^. Chipper allows users to set default parameters for automated segmentation of syllables within each bout of song. We used only the top 2% on signal and used the default settings of 10 ms minimum silence duration and 30 ms minimum syllable duration. Occasionally we adjusted the syllable onsets and offsets (syllable selections) to remove environmental noises or other artefacts from recordings. We subsequently performed Chipper’s song analysis to obtain spectral, song and syllable measurements, from which we selected nine acoustic features of the songs for subsequent analysis (Table S3).

After ensuring none of the acoustic attributes showed strong positive or negative correlation with any others using the ‘ggcorrplot’ function in the R-package ‘GGally’^56^ (Fig. S4), we averaged each spectral measure across multiple recordings of each individual’s song. We then performed discriminant function analysis (DFA) on the average spectral measures using JMP v17.2.0^57^. The DFA contained seven pre-defined groups: the five experimental groups and the control group described above, plus the songs of untutored zoo-bred males from previous years and the typical Blue Mountains wild male reference songs, which included songs of the two wild-origin live tutors.

We then calculated the Mahalanobis distance between each individual’s average song attributes and the centroid of the culturally-dominant typical Blue Mountains reference songs recorded in the wild between 2015 and 2017 and used in the playback tutoring experiments. The Mahalanobis distance represents a measure of acoustic (dis)similarity between the average song of each individual regent honeyeater and the target song of the tutoring experiment^58^. Increasing Mahalanobis distances indicate increasing dissimilarity between an individual’s learned song and the mean of the culturally dominant wild song type.

To assess whether the songs of birds in each treatment group differed from the typical Blue Mountains reference songs (and from those in other treatment groups), we used the Mahalanobis distances described above as the response variable in a generalised linear model (GLM) using the package ‘stats’ in R 4.0.3^59^. The model contained treatment group as a factorial fixed effect and individual age in days at song recording as a continuous fixed effect. We conducted backwards model selection using the ‘stepAIC’ function from the package ‘MASS’^60^, which revealed that removing age as a non-significant predictor improved model fit (AIC = 1.97, Table S4). While this difference is marginal, age showed no significant effect (*p* =.854), and we retained the simpler model based on parsimony principles. We selected a generalised linear model containing only treatment as a factorial fixed effect. We could not include breeding facility or aviary type as random terms due to unavoidable restrictions on the space available within the breeding facilities for experimentation. We assessed the quality of the model by using the ‘simulateResiduals’ function in the ‘DHARMa’ package^61^(Fig. S3) and conducted post-hoc tests of pairwise significance between treatment groups using the package ‘multcomp’ v1.14–17^62^.

### Animal ethics

Research was approved by the Taronga Conservation Society Australia Animal Ethics Committee in January 2020 code 4a/02/20 and conducted under NSW scientific license number SL101580. Research abided by NSW Animal Research legislation and followed best practice in the ARRIVE guidelines.

## Supplementary Information

Below is the link to the electronic supplementary material.

Supplementary Material 1

## Data Availability

Data and R code is available via the Dryad Digital Repository: [http://datadryad.org/stash/share/rI7eUQ3-qgY1hxqiIQ3HsR8tJcpndqLVEN3LZVDGBRc](http:/datadryad.org/stash/share/rI7eUQ3-qgY1hxqiIQ3HsR8tJcpndqLVEN3LZVDGBRc).
