# Supplementary material for: Rescue of the traditional song culture of a critically endangered songbird
Source: Sci Rep. 2026 Feb 25;16:11058. doi: 10.1038/s41598-026-40115-3 (PMC13043855; doi:10.1038/s41598-026-40115-3)
Supplement: Supplementary file 1 — Supplementary Material 1 [file 41598_2026_40115_MOESM1_ESM.docx]

Rescue of the traditional song culture of a critically endangered songbird

**Supplementary Materials**

**Supplementary Text S1 – Study populations and treatment groups**

*Study population*

The regent honeyeater zoo-breeding program was established in 1995 in response to a dramatic decline in the wild population. Nine nestlings collected from the wild formed the original zoo population, with birds subsequently recruited in 1997 (n=2), 2012 (n = 4), 2019 (n = 3) and 2023 (n = 2). To date, ~400 zoo-bred regent honeyeaters have been released into the wild.

Birds in this study were born and housed in Taronga Zoo (TZ), Sydney and/or Taronga Western Plains Zoo (TWPZ), Dubbo. All birds included in this study were part of the active zoo-breeding program and therefore subject to routine husbandry protocols. As is often the case in conservation management, resources for time and space within the breeding facilities were limited. As such, experiments needed to be incorporated in the least disruptive manner to ensure they achieved the overall aim of establishing wild song culture in the zoo population without impacting negatively the breeding capacity of the program.

*Treatment groups*

Treatment 1 - Control: The control tutoring was conducted at TWPZ in the 2020-2021 breeding season. Nine juvenile males were crèched together according to traditional husbandry protocols on the standard timeline described above and in Table S1. Birds were housed in aviary type 2 (Fig. 1a) at the end of a breeding block of aviaries and were unable to see birds in other treatments. A dummy speaker (Bose FreeSpace 360p Series II) was mounted within the aviary but did not emit sound. The only sounds juvenile males in the control group were exposed to were from each other, and from the surrounding environment such as nearby zoo and wild animals and suburban noises such as air and road traffic.

Treatment 2 – Playback only (large cohort): The playback only (large cohort) treatment was conducted at TWPZ in the 2020-2021 breeding season. Nine birds were housed together in an aviary type 2 (Fig. S1a) according to the standard timeline. A composite track consisting of the songs of 25 different wild male regent honeyeaters singing the typical Blue Mountains song type (each with a duration of ~2 seconds) was broadcast to the juvenile birds from sunrise to sunset every day of the experiment from a broadcast speaker mounted within the aviary. Given the regent honeyeater evolved to be a flocking species^51^, we considered 25 individuals as a reasonable estimate of the number of adult tutors a juvenile could have expected to interact with in a post-breeding flock prior to severe population decline. To approximate the natural variation in songbird singing activity^52,53^, songs were emitted at a more frequent rate (~four songs per minute) for the first four hours and last two hours of the day, than during the middle of the day (~one song per minute). The length of the playback track was increased two months into the experiment to reflect the lengthening of the daylight hours during the Austral summer. A description of the process of composing the playback track, playback system parameters and playback track itself can be found in the supplemental text (Text S2 & Text S3).

Treatment 3 – Live tutoring only (large cohort): The live tutoring only (large cohort) was conducted at TZ in the 2020-2021 breeding season. Thirteen juvenile males were housed together in an aviary type 1 (Fig. S1b) according to the standard timeline. Juveniles were moved into an aviary adjacent to one of the two wild-origin adult males that sang the typical Blue Mountains wild song. Juveniles could both see and hear the adult male but could not physically interact with him for the first 2.5 months, during which period the adult male was breeding and would otherwise have shown aggression to juveniles in the same aviary (Regent honeyeater husbandry guidelines, Taronga Zoo, 2013). After 2.5 months, the two aviaries were merged, and the juveniles could directly interact with the adult male.

Treatment 4 – Playback only (small cohort): The playback only (small cohort) was conducted at TWPZ in the 2021-2022 breeding season. Four juvenile males were housed in an aviary type 2 (Fig. S1a) according to the standard timeline and were unable to see birds in adjacent aviaries. All other experimental parameters were as described above for Treatment 2- Playback only (large cohort).

Treatment 5: Live tutoring + playback: The live tutoring + playback treatment was conducted at TZ in the 2021-22 and 2022-2023 breeding seasons. Eighteen juvenile males were housed in two separate aviaries: eight (2021-2022) in a type 1 aviary (Fig. S1d) and 10 (2022-2023) also in a type 1 aviary (Fig. S1d). Birds were moved from their natal aviary into a crèche aviary adjacent to a wild-origin adult male according to the standard timeline. As in treatment 3, juvenile birds were able to both see and hear the adult male but could not physically interact with him for the first 2.5 months until the aviaries were merged. Unlike treatment 3, during the period that juveniles could not interact with the wild-origin adult male, they were exposed to playback in the same way as treatment 4. After the juveniles and adult male were united, the broadcast playback continued until the end of the breeding season.

Treatment 6: Live tutoring only (small cohort): The live tutoring only (small cohort) experiment was conducted at both TZ and TWPZ in the 2022-2023 breeding season. Fourteen birds were housed in three separate aviaries, with five juveniles each in two type 2 aviaries (Fig. S1c) at TZ and four juvenile males in one type 3 aviary at TWPZ (Fig. S1c). In the live tutoring only (small cohort) experiments, juvenile males were transferred from their natal aviary according to the standard timeline to a crèche aviary with a zoo-bred adult male tutor (~18 months old) that had successfully learned the typical Blue Mountains song in the previous season’s Treatment 5 experiment. During the tutoring period they could see, hear and physically interact with the live tutor. In summary, the experiment involved 68 birds: two wild origin males (tutors) and 66 juvenile zoo-bred males, of which four were used as live adult tutors in subsequent years (Table 2).

**Supplementary Text S2 – Composing the Playback Track and Playback systems**

*Composing the playback tracks*

We first gathered 25 recordings of wild-origin males who sang the Typical Blue Mountains song-type. These were sourced largely from the wild using recordings obtained in Crates et. al (2021, n = 23), as well as one recording made by D. A of a wild-origin bird at TZ who sang Blue Mountains typical song-type and one recording from Xeno-Canto.

We initially composed 14 playback tracks. Each day of the week had a unique playback track, and two time periods had a set of seven tracks. The first seven of these time periods were 12 hours in length to fill the average Austral daylight hours between September and November. The second seven were 14 hours in duration, designed to fill the average Austral daylight hours between December and March.

We divided tracks between peak and off-peak periods. The peak period was designed with the intention of mimicking the increased call activity of songbirds early in the morning and late evening periods (Catchpole and Slater, 2008). During the peak period, songs were broadcast at a more frequent rate than the off-peak period during the middle of the day. On our tracks the peak period ran for the first four, and the last two hours of the day, regardless of day length. The off-peak period ran in the warmer, middle part of the day in between peak periods. The off-peak period of the playback track broadcast songs at a less frequent rate than the peak period. Between September and November, the off-peak period was six hours in length. Between December and March, the off-peak period was eight hours in length.

To compose the tracks, we developed two randomisation matrices each for the peak and off-peak periods. The first randomisation matrix was designed to set the order of the song presentation and then the timing of the song presentations. For the peak period, songs were emitted at an average rate of one song per 25 seconds (randomised silence between 12- 75 seconds). For the off-peak period, songs were emitted at an average rate of one song per 93 seconds (randomised silence between 60-180 seconds).

We first post-processed recordings in audacity to remove any unwanted sounds. We then ran a 300Hz High Pass Filter to all songs to reduce low end noise. We then created 10, one-hour long blocks of both peak and off-peak playback, by arranging songs in Apple’s ‘garageband’ according to the randomisation matrix. We created another randomisation matrix to decide the presentation of one-hour blocks to make the peak and off peak tracks for each day. These one-hour blocks were arranged in apple’s ‘garageband’ according to the randomisation matrix to make seven peak periods of four hours (morning) and seven peak periods of two hours (evening). We repeated this to make seven off peak periods of six hours of length, and seven off peak periods of eight hours in length. To compose the final 12- and 14-hour playback tracks we randomly assigned one morning peak period, one off peak period and one evening off peak period to create one track. This was done in Audacity software owing to the extremely large file size that was inappropriate for garage band. Files were exported as high-quality ‘.wav’ files and assigned one day each per week for broadcast.

In 2021/2022 season we increased the song playback rate. To do this we followed all of the above steps this time decreasing the randomised silence period by half (effectively doubling the playback rate).

*Playback systems*

We employed two playback systems throughout the three years. At both zoos the broadcast speakers were Bose ‘Freespace’ outdoor speakers. In TZ we used a XX amplifier connected to a windows computer. Playback was initiated using the ‘Windows Task Scheduler’ software. At TWPZ, where a permanent indoor space was not available, we used a portable set up housed in a waterproof box. We used a Swamp Industries line amplifier connected to an iPod touch. We used the default automation app to schedule playback.

**Text S3 – Rationale for adaptive decisions for song tutoring protocols.**

In the 2020/21 season (year one), we trained one cohort with playback at Taronga Western Plains Zoo (TWPZ), one cohort with live tutors at Taronga Zoo (TZ) and one cohort remained un-tutored as a control group at TWPZ. It was clear that live tutoring resulted in better song outcomes than playback, while playback alone was only marginally better the control group. Songs from the live tutoring only (Large cohort) did appear to be more complex than the playback and control groups, however, the final songs of all groups did not approach the wild reference song from a listener’s perspective and subsequent statistical analysis of acoustic features confirmed this (as reported in the main text). This evidence, combined with observations from researchers and husbandry staff showed that these methods had potential if some minor changes were made to the protocols.

During the first season, we observed that in the playback group, the vocalisations of the juveniles were far more frequent than the playback itself, and that juvenile birds tended to interact and vocalise more often in response to each other than in response to playback emitted from the loudspeakers. We believed that interactions with real birds are more enriching than playback and as such juveniles would preferentially learn from a live model, even if they were also juvenile (i.e., Varkevisser 2022; Derregnaucourt 2013). A similar pattern of behaviour was observed in the live tutor group, whereby juveniles did interact with the tutor, but the volume of juveniles appeared to result in most interactions being with other juveniles instead of the adult tutor. We also noted that for the earliest period of their life, young birds didn’t hear wild song as the tutor was not vocal during his own breeding period. This resulted in the following changes for season 2021/22:

1. Rate of song playback was doubled in the both the playback only and live tutoring cohorts.
2. Adult tutor to juvenile ratio was limited to a maximum of1:5.
3. Playback was introduced during the early phases of the live tutoring cohort. The previous live tutoring cohort in 2021 was not exposed to additional playback.

Given the severity of ongoing population decline in regent honeyeaters and the importance of the zoo-breeding program to their recovery efforts (Heinsohn *et al.,* 2022; Appleby *et al.,* 2024), we prioritised implementing all the above changes at once to maximise the chances of successful song learning over a more rigorous step-by-step experimental approach. This was also in-part driven by a lack of space in the zoo setting, where implementing each experimental change in separate cohorts would have taken several years to deploy.

In 2021/22 the playback only (small) cohort showed little improvement compared with the playback only (large) cohort. This indicated that song learning is likely multimodal (Varkevisser et al. 20220) in regent honeyeaters; that is, interaction with a tutor is critical and as such, we decided to cease playback only cohorts in future years in favour of expanding live tutoring. In the live tutoring + playback cohort, we frequently detected wild-type songs by ear during the song tutoring which was later confirmed statistically and are displayed below in fig. S1.

While the 2021/22 season produced juvenile birds whose songs resembled the reference wild song type, the decision to implement multiple changes to the protocols in one year created ambiguity around whether the smaller cohort or the reinforcement of the tutor with playback was the critical factor determining successful song learning. To disambiguate this, we introduced the live tutoring only (small) cohort.

**Supplementary Tables**

| **Supplementary Table 1** |
| --- |
| Table showing the model estimates of generalised linear model regressions using to formula Mahalanobis distance ~ Treatment + Age |
| \|  \| Mahalanobis Distance \| \| \| \| --- \| --- \| --- \| --- \| \| *Predictors* \| *Estimates* \| *CI* \| *p* \| \| (Intercept) \| 2.56 \| 0.13 – 4.99 \| **0.039** \| \| Control (T1) \| 2.03 \| 0.40 – 3.65 \| **0.014** \| \| Playback (Large, T2) \| 1.22 \| -0.32 – 2.77 \| 0.121 \| \| Live Tutoring Only (Large, T3) \| 2.55 \| 1.16 – 3.94 \| **<0.001** \| \| Playback (Small, T4) \| 2.30 \| 0.90 – 3.70 \| **0.001** \| \| Live Tutoring + Playback (T5) \| -0.33 \| -1.74 – 1.08 \| 0.643 \| \| Live Tutor (Small, T6) \| -0.22 \| -1.75 – 1.30 \| 0.773 \| \| Age \| -0.00 \| -0.01 – 0.01 \| 0.854 \| |

***Supplementary Table 2*** –

Timeline Example 2022/23 season (Live Tutor + Playback). Table shows the timeline for two live tutoring + playback cohorts in the 2022/23 season. Light-blue shading represents the period where the juvenile bird is in the natal aviary with its parents. Pale-yellow represents the period when the bird is in a an experimental creche exposed to playback while next to a wild male with who they can’t physically interact but can see and hear. The pale-green shading represents the period when birds are in the creche aviary with the live tutor as well as exposed to song playback. The dashed red line denotes the addition of the live to tutor to the creche aviary.

| ID | August | | | September | | | | October | | | | November | | | | December | | | | January | | | | February | | | | March | | | |
| --- | --- | --- | --- | --- | --- | --- | --- | --- | --- | --- | --- | --- | --- | --- | --- | --- | --- | --- | --- | --- | --- | --- | --- | --- | --- | --- | --- | --- | --- | --- | --- |
| **WEEK** | 3 | 4 | | 1 | 2 | 3 | 4 | 1 | 2 | 3 | 4 | 1 | 2 | 3 | 4 | 1 | 2 | 3 | 4 | 1 | 2 | 3 | 4 | 1 | 2 | 3 | 4 | 1 | 2 | 3 | 4 |
| MALE | BIRD 1 BORN | |  |  |  |  |  |  |  |  |  |  |  |  |  |  |  |  |  |  |  |  |  |  |  |  |  |  |  |  |  |
| MALE |  | |  |  | BIRD 3 BORN | |  |  |  |  |  |  |  |  |  |  |  |  |  |  |  |  |  |  |  |  |  |  |  |  |  |
| MALE |  | |  |  |  |  |  |  |  | BIRD 4 BORN | |  |  |  |  |  |  |  |  |  |  |  |  |  |  |  |  |  |  |  |  |
| MALE |  | |  |  |  |  |  |  |  | BIRD 5 BORN | |  |  |  |  |  |  |  |  |  |  |  |  |  |  |  |  |  |  |  |  |
| MALE |  | |  |  |  |  |  |  |  |  |  |  |  | BIRD 8 BORN | |  |  |  |  |  |  |  |  |  |  |  |  |  |  |  |  |
| MALE |  | | BIRD 2 BORN | |  |  |  |  |  |  |  |  |  |  |  |  |  |  |  |  |  |  |  |  |  |  |  |  |  |  |  |
| MALE |  | |  |  |  |  |  | BIRD 3 BORN | |  |  |  |  |  |  |  |  |  |  |  |  |  |  |  |  |  |  |  |  |  |  |
| MALE |  | |  |  |  |  |  |  |  |  | BIRD 6 BORN | |  |  |  |  |  |  |  |  |  |  |  |  |  |  |  |  |  |  |  |
| MALE |  | |  |  |  |  |  |  |  |  |  | BIRD 7 BORN | |  |  |  |  |  |  |  |  |  |  |  |  |  |  |  |  |  |  |

**Supplementary Table 3:** Acoustic features measured in ‘Chipper’ and whether they were included in the discriminant function analysis.

| **Feature** | **Included in DFA?** |
| --- | --- |
| Number of Unique Syllables | **Yes** |
| Smallest Syllable Frequency Range (Hz) | **Yes** |
| Average Syllable Lower Frequency (Hz) | **Yes** |
| Largest Syllable Frequency Range (Hz) | **Yes** |
| Number Syllables per Bout Duration (1/ms) | **Yes** |
| Number of Syllables | **Yes** |
| Overall Syllable Frequency Range (Hz) | **Yes** |
| Std. Deviation Syllable Frequency Range (Hz) | **Yes** |
| Smallest Syllable Duration (ms) | **Yes** |
| Average Silence Duration (ms) | **No** |
| Average Syllable Duration (ms) | **No** |
| Average Syllable Duration (ms) | **No** |
| Average Syllables Upper Frequency (Hz) | **No** |
| Bout Duration(ms) | **No** |
| Largest Silence Duration (ms) | **No** |
| Largest Syllable Duration (ms) | **No** |
| Largest Syllable Freq Range (Hz) | **No** |
| Max Syllables Frequency (Hz) | **No** |
| Min Syllables Frequency (Hz) | **No** |
| Smallest Silence Duration (ms) | **No** |
| Std. Dev. Silence Duration (ms) | **No** |

**Supplementary Table 4**

| Table showing results of backwards model selection. Each step removes a predictor and shows the resulting AIC. Text in bold shows the strongest performing model. | | | |
| --- | --- | --- | --- |
| Model Formula | AIC | Predictor to Remove | New AIC |
| Mahalanobis Distance~ Treatment + Age | 264.71 | Age | 262.74 |
|  |  | Treatment | 334.25 |
| **Mahalanobis + Distance~ Treatment** | **262.7** | Treatment | 340.3 |

**Supplementary Figures**


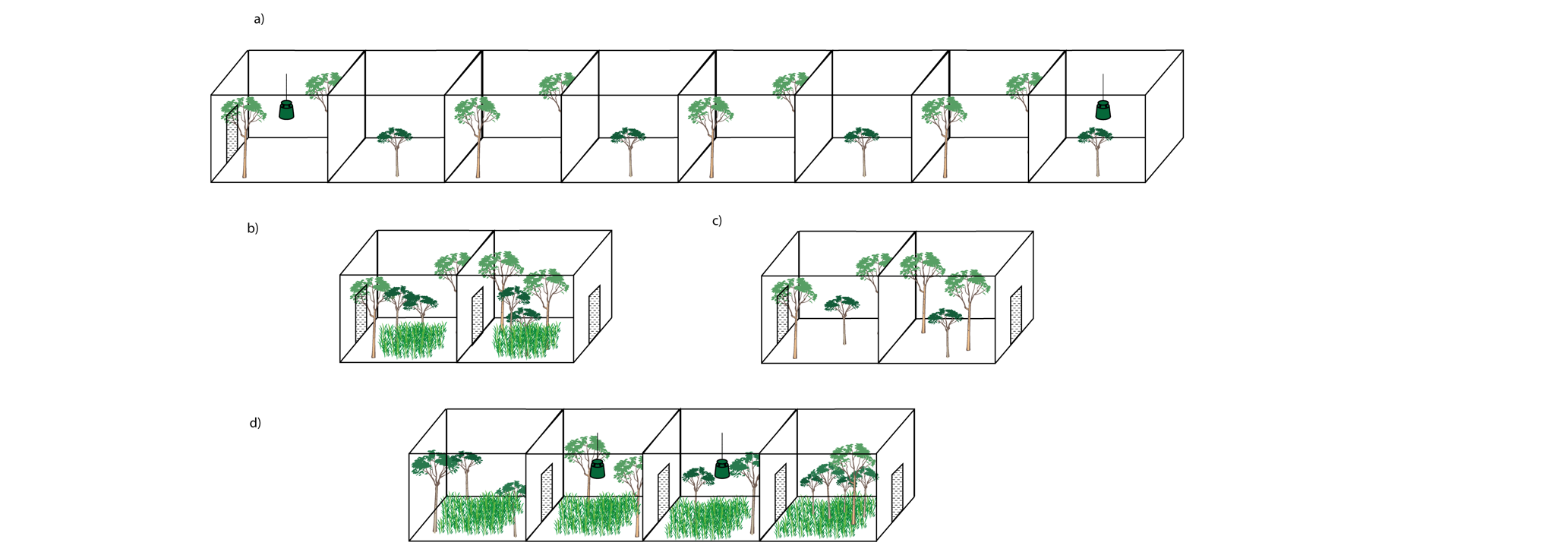


**Fig. S1:** Schematic of regent honeyeater song tutoring aviaries for the different song tutoring protocols. The first, aviary type 1 (a) at Taronga Zoo, (6m x 3.3m x 3m), the second two (b & c) at Taronga Western Plains Zoo, aviary type 2 (4m x 4m x 6m) and type 3 (d) (6m x 4m x 6m). Aviaries were typical of those used as crèche aviaries during normal breeding and post-breeding procedures. A) shows playback only tutoring aviary configuration, where juvenile pupil groups were placed in outer aviaries of an eight-aviary block with broadcast speakers. Breeding pairs housed in the aviaries between. Aviaries were separated by solid dividers preventing visual contact between aviaries. B) shows the configuration of the live tutor only cohort (large) where adult tutor was initially housed in the left aviary while juvenile pupils were housed in the right aviary but could both see and hear the tutor. The tutor was subsequently introduced to the juveniles at the conclusion of the breeding period by opening the adjoining door. C) shows the configuration of the live tutor only (small) cohorts where both the adult tutor and juvenile pupils were housed together in one aviary. Two cohorts were housed side by side with a solid divider between preventing visual contact. D) shows the live tutor + playback configuration where the adult tutor was initially housed in the left aviary while juvenile pupils were housed in the right aviary, but could both see and hear the tutor as well as exposed to broadcast playback. The tutor was subsequently introduced to the juveniles at the conclusion of the breeding period by opening the adjoining door.


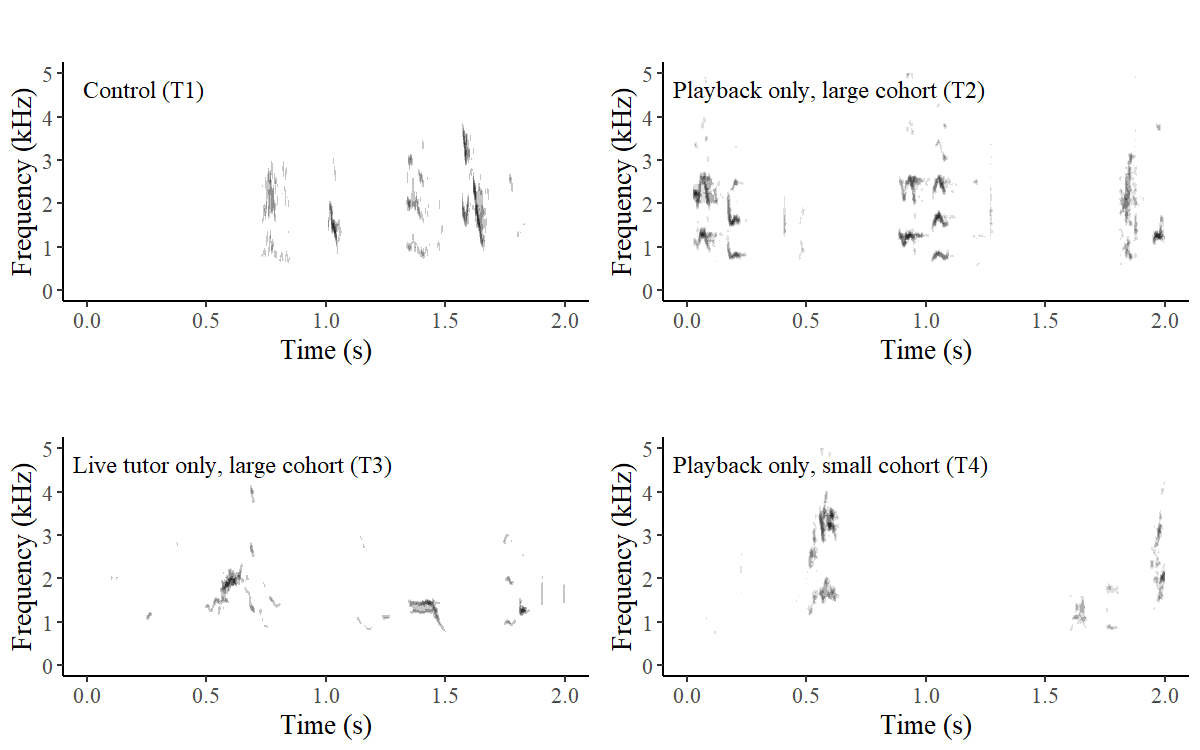


**Fig. S2**: Indicative spectrograms of the songs of juvenile regent honeyeaters included in song tutoring treatment groups 1 – 4.


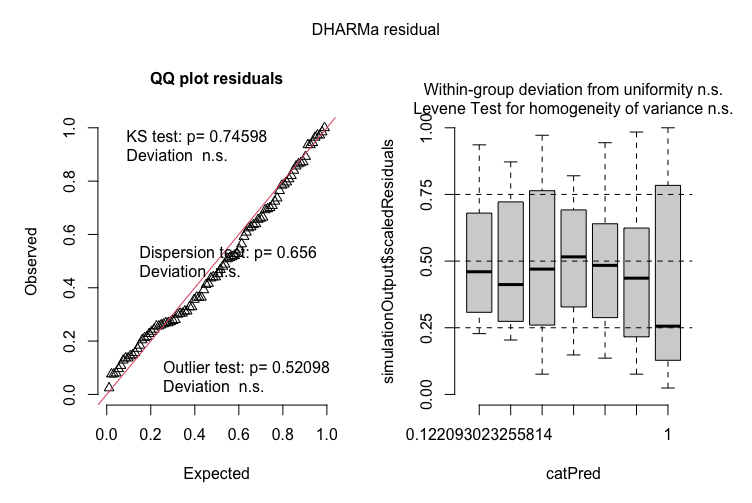


**Fig. S3.** Simulated Residuals of Generalised Linear Regression (Mahalanobis Distance ~ Treatment)


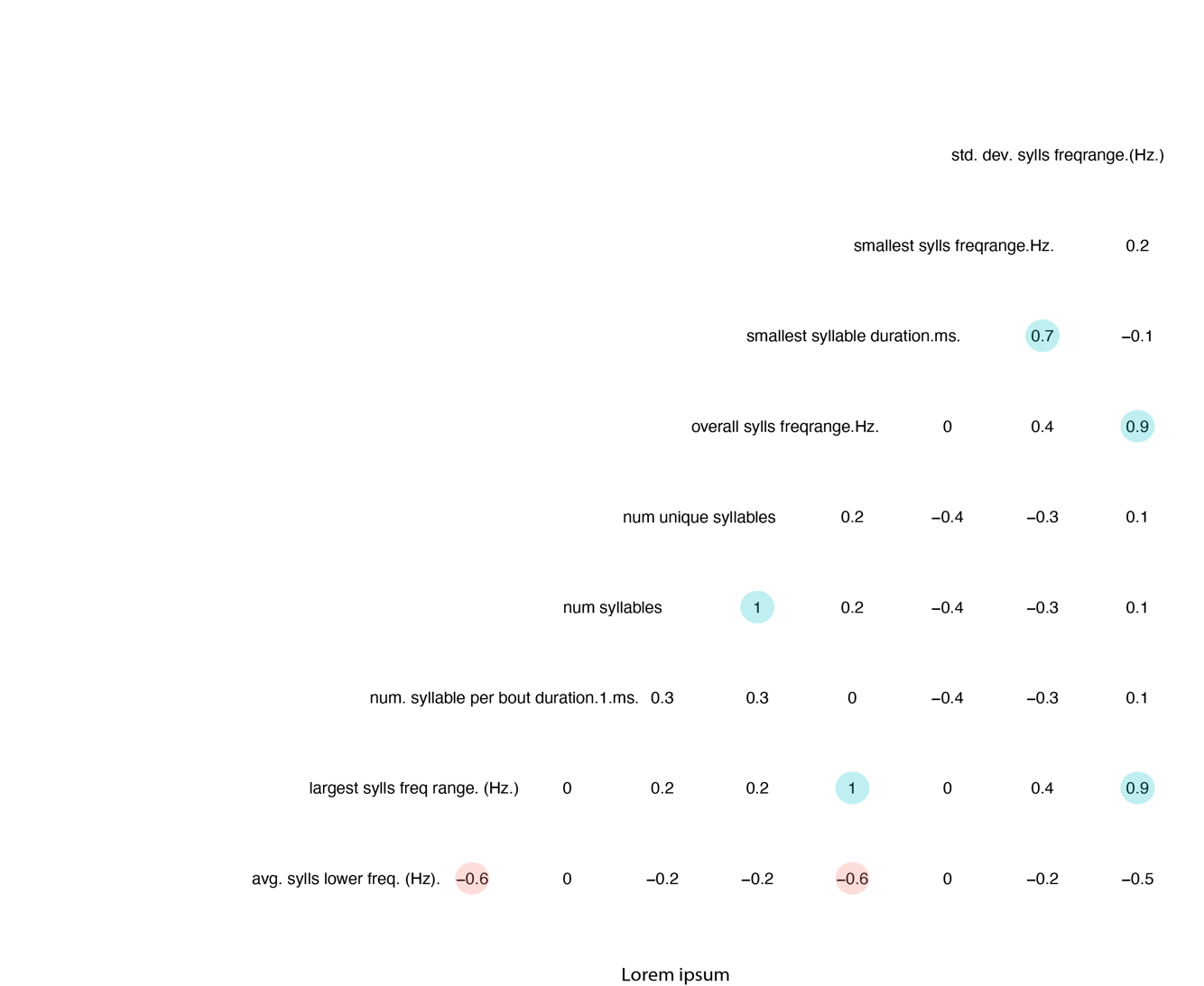


Fig. S4 – Correlation matrix of acoustic features included in discriminant function analysis.
